# Team science: A syllabus for success on big projects

**DOI:** 10.1002/ece3.10343

**Published:** 2023-07-31

**Authors:** Delaney M. Peterson, Sarah M. Flynn, Riley S. Lanfear, Chelsea Smith, Logan J. Swenson, Alice M. Belskis, Stephen C. Cook, Christopher T. Wheeler, Jessica F. Wilhelm, Amy J. Burgin

**Affiliations:** ^1^ Department of Biological Sciences University of Alabama Tuscaloosa Alabama USA; ^2^ Center for Ecological Research University of Kansas and Kansas Biological Survey ‐ Center for Ecological Research Lawrence Kansas USA; ^3^ Department of Biological Sciences Idaho State University Pocatello Idaho USA; ^4^ University of Kansas and Kansas Geological Survey Lawrence Kansas USA; ^5^ Department of Ecosystem Science and Management The Pennsylvania State University State College Pennsylvania USA; ^6^ Department of Biology University of Oklahoma Norman Oklahoma USA

**Keywords:** collaboration, communication, graduate training, research skills, team science

## Abstract

Interdisciplinary teams are on the rise as scientists attempt to address complex environmental issues. While the benefits of team science approaches are clear, researchers often struggle with its implementation, particularly for new team members. The challenges of large projects often weigh on the most vulnerable members of a team: trainees, including undergraduate students, graduate students, and post‐doctoral researchers. Trainees on big projects have to navigate their role on the team, with learning project policies, procedures, and goals, all while also training in key scientific tasks such as co‐authoring papers. To address these challenges, we created and participated in a project‐specific, graduate‐level team science course. The purposes of this course were to: (1) introduce students to the goals of the project, (2) build trainees' understanding of how big projects operate, and (3) allow trainees to explore how their research interests dovetailed with the overall project. Additionally, trainees received training regarding: (1) diversity, equity & inclusion, (2) giving and receiving feedback, and (3) effective communication. Onboarding through the team science course cultivated psychological safety and a collaborative student community across disciplines and institutions. Thus, we recommend a team science course for onboarding students to big projects to help students establish the skills necessary for collaborative research. Project‐based team science classes can benefit student advancement, enhance the productivity of the project, and accelerate the discovery of solutions to ecological issues by building community, establishing a shared project vocabulary, and building a workforce with collaborative skills to better answer ecological research questions.

## Graphical abstract

Team Science is on the rise to address complex ecological issues which cannot be tackled in individual research labs or management programs. Students on big projects often struggle with a clear understanding of the projects goals, their role on the team, project policies and procedures, and knowing how to engage in key scientific tasks such as co‐authoring papers. We recommend that big, collaborative projects begin with formal Team Science courses for trainees, which benefits student advancement, enhances the productivity of the project, and accelerates the discovery of solutions to ecological issues.

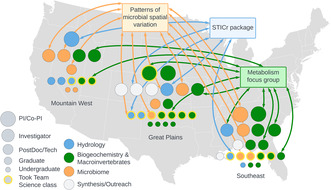

## 1 THE SCIENCE OF TEAM SCIENCE AND TRAINING THE NEXT GENERATION OF SCIENTISTS

Increasingly complex scientific challenges require spanning borders, institutions, and disciplines and thus are inherently infeasible for a single lab group to tackle in isolation. Scientific teams are needed to tackle our most complicated and persistent environmental issues; thus, it is a key skill to build in the future ecological and environmental sciences workforce (Cheruvelil & Soranno, 2018). *Team science* is the term used to denote collaborative scientific research “conducted by more than one person in an interdependent fashion, including research conducted by small teams and larger groups” (National Research Council, 2015). Team science approaches are on the rise in academic research (Farrell et al., 2021; Jones et al., 2008). Training on big (>10 people; National Research Council, 2015), interdisciplinary projects is a great asset to graduate students and early‐career researchers, as large professional networks and collaboration skills are key to building modern science careers (Bennett & Gadlin, 2012; Hampton & Parker, 2011; Pannell et al., 2019; Read et al., 2016).

As scientific teams grow (Wuchty et al., 2007), they become increasingly diverse in terms of disciplinary expertise (O'Rourke et al., 2019), demographics (Gibbs et al., 2019), and geographic and institutional representation (Jones et al., 2008; among other axes of diversity). Increasing diversity on teams is related to innovation and novelty (Hofstra et al., 2020; Yang et al., 2022) and greater impact through increased citations (AlShebli et al., 2018; Freeman & Huang, 2014). However, increasing team science and diversity also leads to an increase in the number of barriers that must be overcome to effectively collaborate (Bennett et al., 2018). Team climate, defined as “the perceived set of norms, attributes, and expectations on a team” affects the team's outcomes (Settles et al., 2019). Thus, understanding how collaborative teams operate and how they can become better is the focus of the emerging field, the Science of Team Science (SciTS), established in 2006 (Hall et al., 2018).

A key report by the National Research Council on the *Enhancing the Effectiveness of Team Science* (National Research Council, 2015) highlights seven key challenges to conducting team science, including: (1) addressing team diversity, (2) integrating across knowledge domains, (3) larger team sizes that create coordination issues, (4) misaligned goals within the team, (5) permeable boundaries and changing team membership, (6) geographic dispersal and coordination, and (7) interdependence on tasks among subgroups. The report also articulates key areas of focus for improving team effectiveness, including: (1) a shared understanding of team processes, (2) university processes to support team science, (3) collaboration technology and virtual collaboration, and (4) the role of funders in requiring teams to articulate collaboration plans. The NRC Report's Chapter 3 concludes that training interventions (e.g., professional development, promoting a shared understanding of roles and goals) are a promising way to increase team effectiveness (National Research Council, 2015). Despite this conclusion and the rise of large collaborative teams conducting science on important issues, professional development training in team science has not kept pace with how science is conducted.

Herein, we present one model of how a project‐specific team science class oriented to the trainees on a big, collaborative project can address many of the challenges highlighted within the NRC report while enhancing student engagement and inclusion. Specifically, this class addressed Challenge 1 by specifically covering diversity and power dynamics and creating a collaborative Code of Conduct (Burgin, 2023a, 2023b). Furthermore, we addressed Challenge 2 by integrating knowledge across domains through the development and workshopping of conceptual models (Panel 1). We spent considerable class time addressing Challenge 3 (coordination), Challenge 4 (goals), and Challenge 6 (geographic dispersion) through a focus of half of the class time on project‐specific documents and policies (Burgin, 2023a). We will describe the details of the class, team‐building activities, and project policies herein with the goal of providing a template for other large collaborative projects to use in building their own cohort‐based team science classes.

**PANEL 1:**
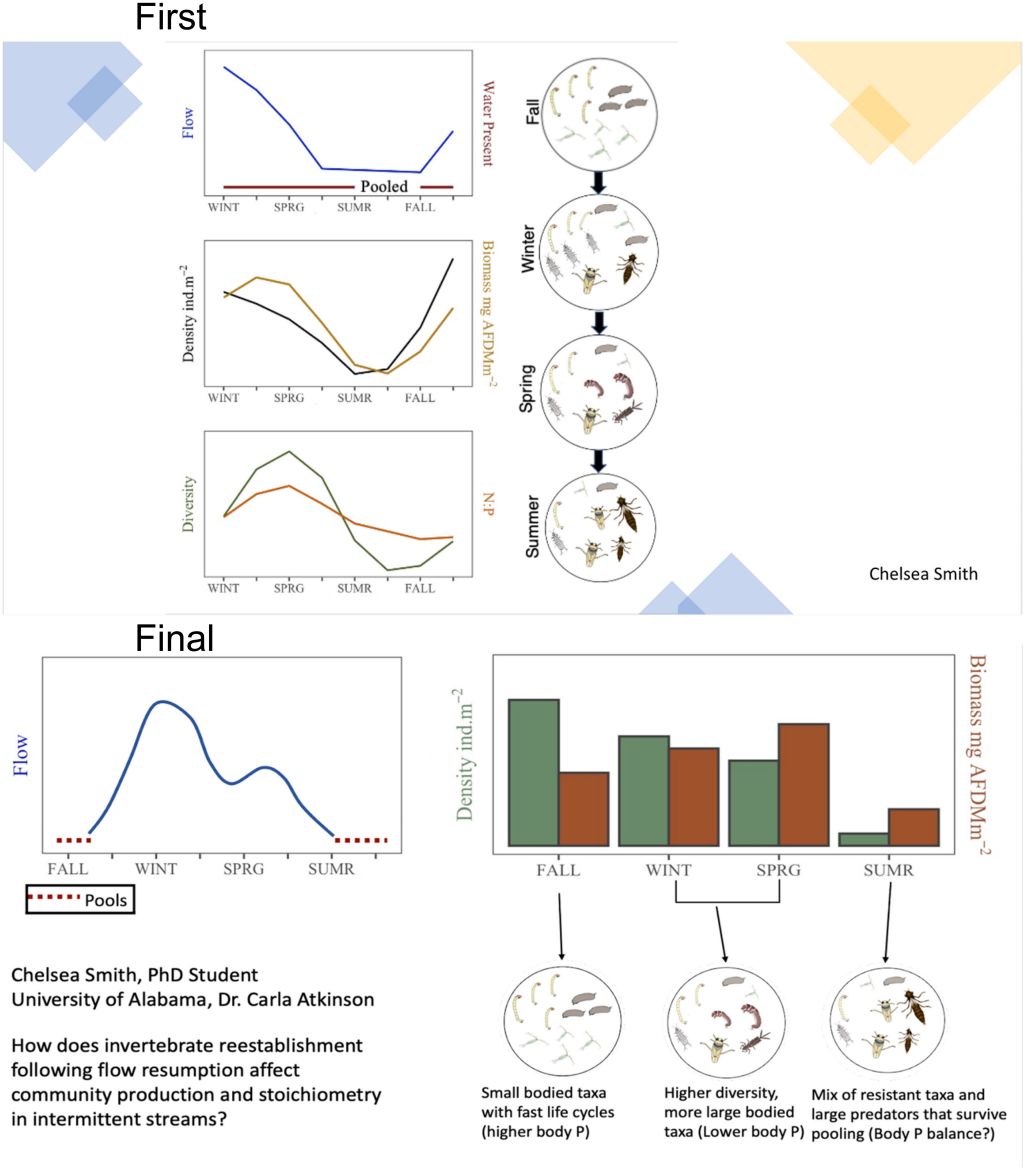
Panel 1 illustrates an example of a conceptual model from a team science student. This student is interested in the effects of flow intermittency on stream macro‐invertebrates. In the first draft (top), the student created a series of panels describing how flow conditions might affect macro‐invertebrate density, biomass, diversity and nutrient stoichiometry throughout the year. The diagrams on the right detail the specific communities corresponding to these changes. Following discussions with other trainees and the instructor, the final draft (bottom) was revised to more clearly show the relationship of flow conditions throughout time with the predicted macro‐invertebrate density and biomass. The community composition panels were simplified, corresponding to each time period with descriptions of the major taxa traits that would contribute to changes in invertebrate stoichiometry.

## 2 CHALLENGES WITH INTEGRATING TRAINEES INTO BIG PROJECTS

Graduate student onboarding and training strategies tailored to team science projects are generally insufficient and sparse (Bennett et al., 2018; Team Science Training, n.d.). Yet, big projects present distinct challenges to students who have not yet developed the skills needed for successful scientific collaboration. Regardless of project size or training program, entering graduate school means conducting research while adapting to a new advisor's management style and navigating the unwritten rules of graduate school, that is, the hidden curriculum (Pensky et al., 2021). Trainees involved in big, interdisciplinary projects must then balance these new skills with challenges, such as working with an extended network of additional advisors with unique personalities, interdependent data streams, unfamiliar methodology, and sometimes conflicting research objectives (Cheruvelil et al., 2014). The approach to ecological research has also shifted in recent years, with more projects adopting a wider lens to gain a more comprehensive view of their ecosystem of interest. Unless intentional effort is made to harmonize the differences among research groups and align research goals, the consequences of poor collaboration frequently fall on graduate students, who are least equipped to deal with them (Deng et al., 2022; Pannell et al., 2019; Read et al., 2016; Zucker, 2012).

A project‐wide, cohort‐based team science course is an ideal vehicle for onboarding trainees to big projects. Our model team science course cultivated community among trainees and gave trainees (the authors of this paper) the skills necessary to collaborate effectively across big projects. Team science classes can orient an emerging team to the design, implementation, and procedures of a specific project. This framework brings students together with the goal of building community on the project, improving communication, providing iterative feedback, and placing individual research into the context of the larger project.

**FIGURE 1:**
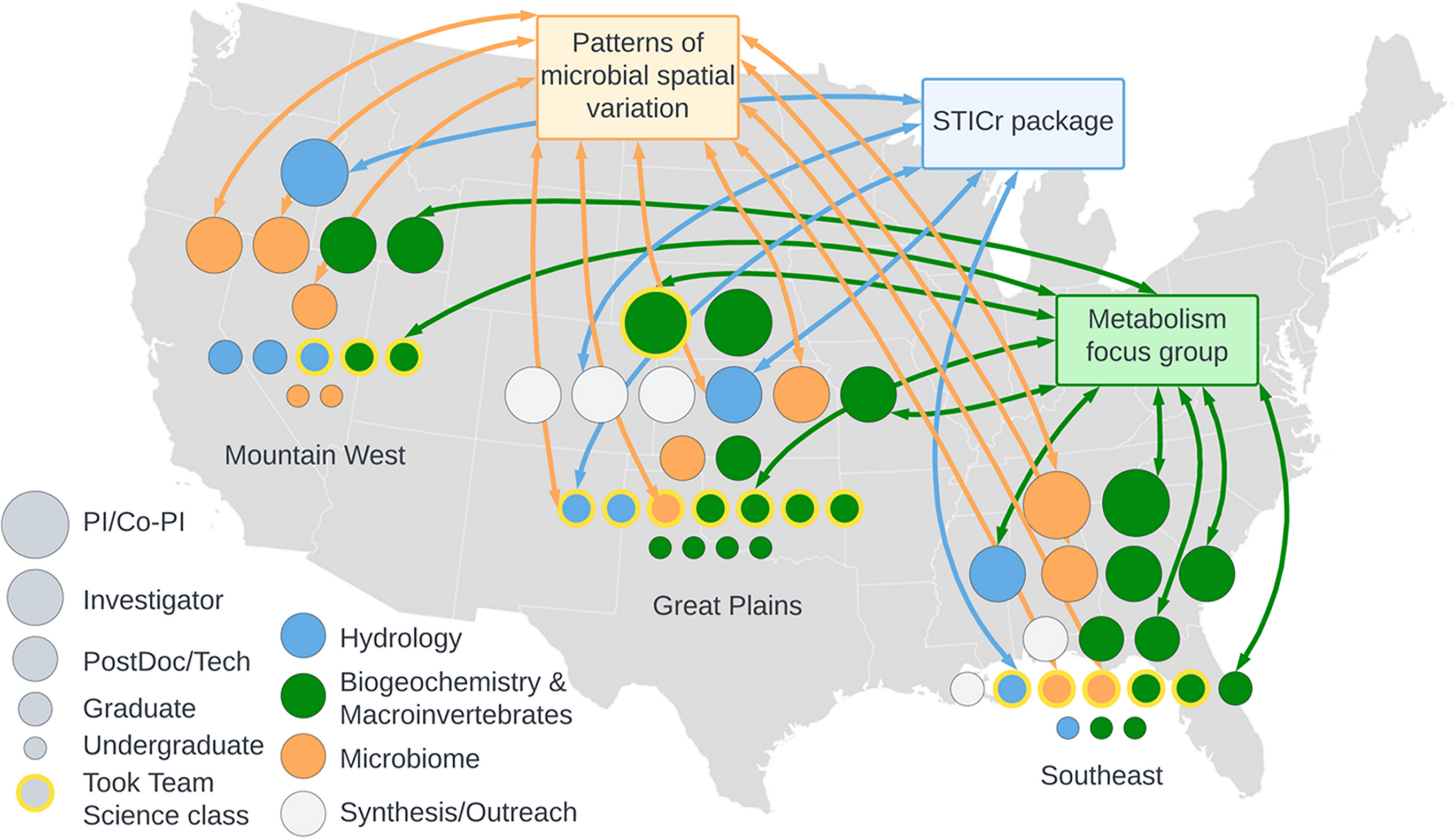
Cross‐disciplinary (color‐coded circles) and cross‐regional collaborations on the Aquatic Intermittency effects of Microbiomes in Steams [AIMS] project were accelerated by the team science course. We highlight one representative collaborative effort lead within each of our three research themes (hydrology, biogeochemistry and macroinvertebrates, and microbiome) that is harnessing expertise from different professional levels and regions on the AIMS project.

Our model course brought together one cohort of trainees from six institutions who were joining a big, interdisciplinary scientific team: the Aquatic Intermittency effects of Microbiomes in Streams [AIMS] project. AIMS is a $6.6 M National Science Foundation [NSF]‐funded collaboration of 19 faculty investigators (~50% early career researchers [ECRs]), six postdoctoral associates, 19 graduate students, and nine undergraduate students spread across three general regions of the US (Figure 1). AIMS applies collaborative, interdisciplinary approaches to study how stream flow intermittency (i.e., periods of drying and wetting) affects downstream water quality. The project includes researchers from various scientific backgrounds, including biogeochemistry, macroinvertebrate ecology, hydrology, and microbiology (Figure 1).

## 3 DESIGNING AIMS' TEAM SCIENCE CLASS

To onboard students to AIMS, Principal Investigator (PI), Dr. Amy Burgin developed a project‐wide, synchronous, two‐credit course in team science. The team science course was designed to balance foundational collaboration principles with details of AIMS‐specific policies and procedures (see syllabus in Burgin, 2023a). Dr. Burgin designed the class based on the organization of the NIH Field Guide to Team Science (Bennett et al., 2018) such that approximately half of the content centered on key skills needed to develop a well‐functioning team. These included Diversity, Equity & Inclusion (DEI) training, constructive and iterative feedback, and effective communication, and were driven by trainee‐led discussions (Bennett & Gadlin, 2012; Burgin, 2023a; Cheruvelil et al., 2014; Cheruvelil & Soranno, 2018). The other half of class time focused on AIMS‐specific documents, including the Code of Conduct (Burgin, 2023b), project proposal, mentoring agreements (Burgin et al., 2023a), authorship policies (Burgin et al., 2023b, 2023c), and project evaluation plan. The project policies, which were the foundation for much of the course, were established through the creation of a project‐wide implementation plan and were therefore already agreed upon by all senior members of the project.

**FIGURE 2:**
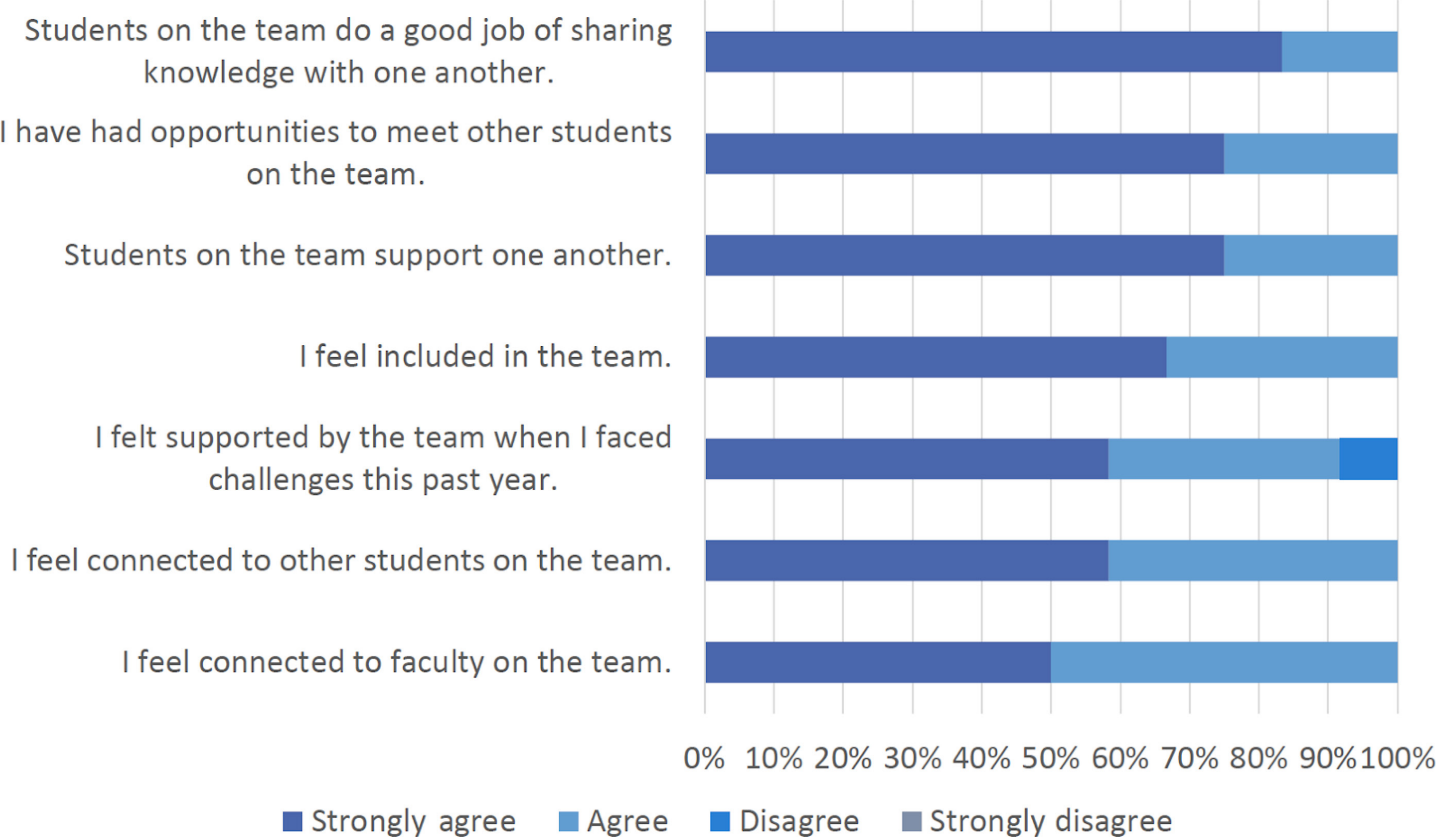
Team science course participants (*n* = 12 respondents, representing 80% of the 15 total students who were enrolled) were assessed based on their feelings of inclusion and connectedness to other students and other AIMS team members. Data provided by Dr. Eric Welch, external evaluator of the AIMS project.

Fifteen graduate trainees from six institutions comprised the cohort of trainees on the project and participated in the team science course offered in Fall 2021. The group met virtually twice a week for 15 weeks with the overarching goal to acquaint new trainees with each other and the AIMS project's policies and procedures (Burgin, 2023a). Virtual class meetings were hosted over Zoom, which allowed us to record classes for future trainees. Course files and discussion boards were hosted with Perusall in the absence of a common Learning Management System (e.g., Blackboard or Canvas). Slack was used for class communication, which fostered open, transparent communication wherein the trainees' advisors could observe and engage in class activities. Trainees, along with the PI, participated in creating a class code of conduct regarding course participation and how to treat one another (Burgin, 2023b). Overall, the team science course assisted trainees with understanding the expectations of their direct advisors and their role within the larger project, while also building a community among the graduate students and promoting feelings of inclusion and support on the team (Figure 2).

The major product for student assessment was the development of conceptual models by each trainee specific to their individual research questions in relation to the overall project goals and datasets. Building and workshopping conceptual models of their study systems, and thinking through the connections to the larger project, encouraged each trainee to envision their role within the overall team and assisted in building connections among sub‐projects within the overall AIMS network. Iterative feedback on the conceptual models was provided by small groups throughout the course (assignment details and an example provided in Panel 1). These small groups were intentionally designed to be cross‐disciplinary and cross‐regional, allowing trainees to develop relationships with others they may not have interacted with otherwise, in addition to helping build a working project vocabulary that reached across disciplines. Additionally, trainees had the opportunity to meet one‐on‐one with the PI to receive individualized feedback. This culminated in the course final in which trainees presented their conceptual models at the (virtual) AIMS project All Hands Meeting, whereby they formally introduced themselves and their ideas to the larger project team. Through sharing these conceptual models with each other and the AIMS team, trainees learned where their research fits in the larger context of the AIMS project. The exercise also facilitated the introduction of trainees to the network of collaborators. While sharing these conceptual models, students were able to gauge where there was overlap and potential collaboration between different fields of study. This led to trainees working together to identify how certain ecosystem processes could be working in tandem and how best to quantify those interactions.

## 4 WHAT TRAINEES LEARNED FROM AIMS TEAM SCIENCE

Team science allowed the cohort to develop psychological safety, which ultimately enabled effective team functionality within the larger AIMS project. Psychological safety is the shared belief that the team is a safe space for risk‐taking and exchanging new scientific ideas (Edmondson, 1999). This is especially important for trainees and early career researchers, who often face underdeveloped confidence and general inexperience, making it more difficult to participate in large projects with unfamiliar collaborators. Developing psychological safety early on allowed AIMS trainees to freely share ideas by establishing trust among team members. Our team science class provided a safe space to gain confidence, get to know each other, and receive feedback from peers. This resulted in increased feelings of being supported by, connected to, and included in the project team (Figure 2). Additionally, the incorporated DEI training [provided by the ADVANCEGeo Network] educated and empowered team members in building inclusive workspaces. Ultimately, a focus on fostering psychological safety and trust among trainees fostered collaboration across the AIMS project.

Onboarding via the team science class enabled AIMS trainees to understand how a big project operates and adjust to new expectations. The practical operation of a big, interdisciplinary project poses challenges for trainees to navigate due to differences in disciplinary jargon, best practices, and acknowledged goals. Furthermore, the challenges of managing personal interactions, competing agendas, and demands on time increase as a function of team size. To address these challenges, the team science class discussed the AIMS project's logistics and policies (e.g., Burgin et al., 2023a, 2023b, 2023c), thereby building a common vocabulary and understanding of project‐wide goals. Further, this allowed trainees to advance their knowledge base beyond specific sub‐disciplines. This course introduced trainees to each other despite disciplinary and institutional barriers, facilitating effective communication and building a sense of community. Discussions of project expectations were provided to the group at the same time and in the same format, leaving less room for misinterpretation or miscommunication.

The course launched early and open conversations about authorship, data sharing, and workload distribution. Notably, AIMS trainees valued discussing the authorship policy (Burgin et al., 2023b, 2023c; modeled on policy described in Cheruvelil et al., 2014), as it gave a clear outline of what was expected from project members as co‐authors on papers and talks. AIMS' internally posted authorship memos (Burgin et al., 2023c) allowed other collaborators to express interest in co‐authorship and determine non‐advisors' roles on our papers. Thirty‐seven authorship memos have been written so far to further a variety of products and manuscripts, both within and across the regions and institutions in AIMS (Figure 1). Examples include Mountain West and Southeast regions' microbial ecologists working with Great Plains hydrologists to describe microbial diversity in the Konza Prairie (Figure 1). Hydrology collaborators across all regions are building a R package for processing flow intermittence sensor data (Figure 1). A subset of biogeochemists across all regions are focused on stream metabolism (Figure 1). By demystifying the authorship process early on, trainees were empowered to lead 68% of all products so far, allowing us to share our findings and build strong networks early in our careers. Additionally, the AIMS Mentorship Agreement (Burgin et al., 2023a) provided similar guidance across labs and institutions by facilitating discussions between faculty mentors and trainees of mutual expectations. These discussions led to clarity and transparency in trainees' relationships with advisors and other project mentors, while also providing a foundation for continuing the conversations after the class was over. Overall, setting these expectations early allowed us to understand our role more clearly on a big project with many competing demands.

Collaboration requires building skills in effective group communication including integrating layers of feedback, building awareness of disciplinary jargon, and navigating misunderstandings before they become conflicts. To practice these skills, trainees received in‐class feedback on our conceptual models from AIMS peers, thereby building confidence before presenting to more experienced researchers. By the time trainees reached the high‐stakes environment of a full‐project meeting, the ideas had been vetted by our peers, mentors, supervisor, and course instructor. Iterative feedback improved trainee's individual conceptual models (Panel 1) and gave us the opportunity to collaborate across disciplines, allowing us to begin our thesis and dissertation work with many levels of interdisciplinary feedback within our first semester. Additionally, it is inevitable that conflicts will arise when many distinct disciplines and personalities are represented on big projects; however, team science provided a scaffolding to navigate points of friction. This ensured that conflicts did not turn into outright disputes, but instead led to greater understanding between collaborators. For AIMS, a focus on building group communication and conflict management skills allowed us to more confidently navigate the challenges inherent in working with an interdisciplinary team.

## 5 COMMUNITY AND COLLABORATION FOSTERS EFFECTIVE TEAM SCIENCE

Without the team science course, jumping headfirst into a big project would have been overwhelming and isolating. AIMS' team science class established a community among trainees despite physical and disciplinary boundaries. Improving collective communication skills enabled trainees to collaborate across disciplines on ecological issues like identifying drivers of water quality and quantity. The class empowered trainees to become more involved in the AIMS project as we have learned to confidently articulate ideas and effectively incorporate feedback. These skills helped trainees navigate intense, cross‐regional sampling efforts, as well as sustain engagement through virtual collaborations, such as our metabolism focus group. In addition, taking time for a deeper discussion of the proposal allowed trainees to understand the logistics and management involved in running a big project, which is an essential skill to build in modern scientific careers.

The community AIMS trainees built in the team science course continued after the class; the student Slack space remains an active resource for skill sharing among peers, and trainees meet monthly for coffee‐talk about the project and graduate school. The class helped trainees get to know each other and the project goals, thereby enabling us to make valued contributions to the project, and to reach across regions and disciplines to collaborate with other trainees. This is demonstrated by 25 cross‐region student‐led authorship memos on diverse topics such as partitioning baseflow sources in non‐perennial streams, quantifying stream metabolism, examining biogeochemical response to storm events, and developing an open‐source software package for flow intermittency loggers.

While this cohort‐based, project‐specific team science class is one model for student training, we also recognize that other models and interventions exist for incorporating team science training into student development. For example, the National Socio‐Environmental Synthesis Center (SESYNC) builds team science training into the Graduate Pursuit program (Wallen et al., 2019). Learning modules, activities, and competencies have been developed and can be incorporated into project workshops and team‐building activities (Gosselin et al., 2020; Pennington et al., 2020). Key tools for those seeking to embark on team science training include the NRC report (National Research Council, 2015), the NIH Field Guide to Collaboration (Bennett et al., 2018), training workshops available through societies such as the American Institute of Biological Sciences (AIBS), and the NSF‐supported Toolbox Dialog Initiative (https://tdi.msu.edu/). Further, while we suggest that the cohort model is the ideal vehicle for providing clear and coordinated onboarding, there are ways to adapt this team science course to other big projects. Given that the overarching learning objective is to educate trainees on the principles and tools for team science we have outlined here, this course could be delivered with several different models. In order to address either large distances between institutions or trainees joining the project at different times, the course could be provided asynchronously as a series of recorded lectures. Further, if course meetings or lectures are recorded, these resources could be provided to trainees that join the project after the course was offered. The flexibility of the resources and content we describe will allow other big projects to adapt a team science course to fit their specific needs.

We recommend that big, collaborative projects begin with formal team science courses for trainees, which benefits student advancement, enhances the productivity of the project, and accelerates the discovery of solutions to ecological issues. The positive impacts of the team science course were seen from new team members to established investigators. Team science provided coordinated onboarding, which ensured we understood our role on the project and empowered us to explore our own hypotheses early on, accelerating the pace of our research. As a result, we returned to our lab groups with enhanced training for supportive lab culture, helping PIs build and maintain effective research lab groups of their own. Additionally, project‐wide collaboration was cultivated by demystifying the authorship policies and expectations associated with the project from the start, enabling us to participate in publishing materials with interdisciplinary teams. We summarized the main features of our team science course, as well as the advantages, challenges, and key resources in Table 1 as well as in several of our course and project documents (Burgin, 2023a, 2023b; Burgin et al., 2023a, 2023b, 2023c). Early relationship building translated into a unified sense of team identity, which has been integral to the AIMS' success in launching as a large, collaborative project. Our goal in documenting our experiences and providing our resources is to make it easier for other big, interdisciplinary teams to use this as a scaffolding to build their own team science onboarding programs incorporating trainees to large, interdisciplinary projects.

**TABLE 1:** A summary of the key AIMS team science course attributes, their advantages, challenges, and key resources. These describe the features of the team science course that were most beneficial to trainees and are easily implemented into other courses.

Attribute	Advantages	Challenges	Resources and examples
Cohort model	Building community, well‐trained students	Recruitment, turnover	Social media, project mentors, and advisors, Cohort Building Toolkit, Imperial College of London (n.d.)
Synchronous, for‐credit class	Accountability, psychological safety, networking, iterative feedback	Scheduling across time zones/university calendars	Zoom, Perusall, Syllabus Burgin (2023a)
Introducing extended team members (e.g., Project Ombuds, Data Manager, other collaborators)	Networking, learning different skills, introduction to Ombuds contact for disputes	Getting others to volunteer their time	ADVANCEGeo Bystander intervention training
Reading the project proposal and policies	Understanding project operations; building grant writing	Student engagement	See citations for AIMS‐specific documents
Student‐led discussions	Creates ownership, builds teaching/communication skills	Psychological safety should be established before students present; uneven pressure on introverted students	Team science Syllabus Burgin (2023a)
Class Code of Conduct made with instructor and students	Creates ownership of the class, accountability, respect, confidence, psychological safety	Participation in creation and continued acknowledgment of the Code	Team science Code of Conduct Burgin (2023b)
Introduction of students' ideas to entire project team	Networking, feeling included in the project, building confidence	Scheduling; stage fright; hard to provide meaningful feedback with simultaneous presentations	Conceptual model presentations (Panel 1)
One‐on‐one meeting with instructor and office hours	Creates a relationship between each student and PI; builds psychological safety; practices feedback skills	Scheduling across time zones and competing demands	Zoom, scheduling programs, Team science syllabus Burgin (2023a)

## AUTHOR CONTRIBUTIONS

**Delaney M. Peterson:** Investigation (equal); methodology (equal); supervision (equal); writing – original draft (equal); writing – review and editing (equal). **Sarah M. Flynn:** Investigation (equal); methodology (equal); writing – original draft (equal); writing – review and editing (equal). **Riley S. Lanfear:** Investigation (equal); methodology (equal); writing – original draft (equal); writing – review and editing (equal). **Chelsea Smith:** Investigation (equal); methodology (equal); writing – original draft (equal); writing – review and editing (equal). **Logan J. Swenson:** Investigation (equal); methodology (equal); writing – original draft (equal); writing – review and editing (equal). **Alice M. Belskis:** Investigation (equal); methodology (equal); writing – original draft (equal); writing – review and editing (equal). **Stephen C. Cook:** Investigation (equal); methodology (equal); writing – original draft (equal); writing – review and editing (equal). **Christopher T. Wheeler:** Investigation (equal); methodology (equal); writing – original draft (equal); writing – review and editing (equal). **Jessica F. Wilhelm:** Investigation (equal); methodology (equal); writing – original draft (equal); writing – review and editing (equal). **Amy J. Burgin:** Conceptualization (equal); investigation (equal); methodology (equal); project administration (equal); resources (equal); supervision (equal); writing – original draft (equal); writing – review and editing (equal).

## CONFLICT OF INTEREST

The authors declare no conflicts of interest with regard to this work.

## Data Availability

Data sharing not applicable to this article as no datasets were generated or analyzed during the current study.
